# Safety and efficacy of topical vs intracanalicular corticosteroids for the prevention of postoperative inflammation after cataract surgery

**DOI:** 10.1097/j.jcrs.0000000000000963

**Published:** 2022-05-09

**Authors:** Amy Q. Lu, Monica Rizk, Tara O'Rourke, Kristin Goodling, Erik Lehman, Ingrid U. Scott, Seth M. Pantanelli

**Affiliations:** From the Department of Ophthalmology, Penn State College of Medicine, Hershey, Pennsylvania (Lu, Rizk, O'Rourke, Goodling, Scott, Pantanelli); Department of Public Health Sciences, Penn State College of Medicine, Hershey, Pennsylvania (Lehman, Scott).

## Abstract

To compare the safety and efficacy of topical prednisolone and intracanalicular dexamethasone ophthalmic insert for the prevention of postoperative inflammation after cataract surgery.

**Setting:**

Penn State College of Medicine, Hershey, Pennsylvania.

**Design:**

Retrospective consecutive case series.

**Methods:**

Patients scheduled for elective phacoemulsification cataract surgery with a plan to receive inflammation prophylaxis with topical prednisolone (prednisolone acetate 1 mg/1 mL) between January 2018 and November 2019 or intracanalicular dexamethasone (Dextenza, 0.4 mg) between December 2019 and March 2021 were screened. Patients were seen 1 day, 1 week, and 4 to 16 weeks postoperatively. Medical records were also reviewed for any urgent messages between visits. Primary end points were proportion of eyes with (1) breakthrough inflammation requiring escalation of anti-inflammatory therapy and (2) intraocular pressure (IOP) increase ≥10 mm Hg at 4 to 16 weeks of follow-up. Secondary end points included incidence of intraoperative complications, cystoid macular edema, and infectious sequelae.

**Results:**

358 patient charts (358 eyes) were screened. Of these, 262 eyes of 262 patients met the criteria for inclusion in the study; 131 eyes received topical drops, and 131 eyes received the intracanalicular insert. Among eyes that completed follow-up, 9 eyes (6.9%) in the drops group and 12 eyes (9.2%) in the insert group experienced breakthrough inflammation necessitating treatment (*P* = .50). 2 eyes in the drops group and 1 eye in the insert group had elevated IOP.

**Conclusions:**

Postoperative inflammation prophylaxis with the intracanalicular insert may be associated with similar rates of breakthrough inflammation and IOP elevation as topical drops.

## Abstract 2

Intracanalicular dexamethasone may have similar efficacy as topical corticosteroids in prevention of breakthrough inflammation after cataract surgery and was not found to be associated with an increased risk for IOP elevation.

Cataract surgery is one of the most common ambulatory procedures performed in the United States.^1^ Complications, although rare, include postoperative inflammation, cystoid macular edema (CME), and elevated intraocular pressure (IOP), among others.^2–4^ One standard-of-care option for prophylaxis against postoperative inflammation after cataract surgery is topical steroid eye drops, alone or in combination with topical nonsteroidal anti-inflammatory drugs (NSAIDs). However, topical drops can be challenging to administer correctly. One study showed that 92.6% of patients administered drops incorrectly, despite only 31% of these patients reporting subjective difficulty, implying a discrepancy even among compliant patients.^5^ Furthermore, most patient populations undergoing cataract surgery are elderly, and some may not have the physical capacity or support of others to help correctly instill the drops.^6^

Dropless cataract surgery involves the intraoperative administration of steroid-containing agents to circumvent the need for compliance with a postoperative drop regimen. Several approaches, including sub-Tenon, subconjunctival, intracameral, and intravitreal injection of anti-inflammatory agents, to replace the need for topical anti-inflammatory drops, have been trialed.^7^ In general, these methods were successful in their ability to dampen postsurgical inflammation, in some cases achieving comparable outcomes to those of topical steroid drops. However, these approaches often involve local compounding of the pharmacologic agent and/or intraocular delivery of the drug.

Dextenza (Ocular Therapeutix, Inc.) is a resorbable drug-eluting insert carrying 0.4 mg of dexamethasone intended for insertion into the canaliculus. It is the first intracanalicular insert approved by the U.S. Food and Drug Administration (FDA) to treat inflammation and pain after ophthalmic surgery. In one prospective multicenter randomized clinical trial, it resulted in less anterior chamber cell and postoperative pain after cataract surgery compared with placebo.^8^ Since its FDA approval in 2019, it has also been used following laser-assisted in situ keratomileusis, photorefractive keratectomy, refractive lens exchange, and pars plana vitrectomy with better subjective reports of patient experience and similar levels of postoperative inflammation and pain compared with topical steroid drops.^9–12^ To the authors' knowledge, there has been no direct comparison of Dextenza with a control group receiving topical steroid drops after cataract surgery. The purpose of this study was to compare the safety and efficacy of topical prednisolone acetate and intracanalicular dexamethasone ophthalmic insert for the prevention of postoperative inflammation after cataract surgery.

## METHODS

The study protocol was granted Institutional Review Board (IRB) approval by the IRB of the Pennsylvania State University College of Medicine. The study adhered to the tenets of the Declaration of Helsinki, and HIPAA regulations were followed. The study was designed as a retrospective consecutive case series of patients who underwent planned extracapsular cataract extraction by phacoemulsification with implantation of a posterior chamber intraocular lens by a single surgeon between January 2018 and March 2021. All patients seen by surgeon SMP between January 2018 and November 2019 received topical prednisolone as the primary mode of inflammation prophylaxis, and patients seen between December 2019 and March 2021 were offered the intracanalicular insert as the primary mode of inflammation prophylaxis, provided it was covered by their insurance. Specifically, Medicaid and self-pay patients were excluded because of lack of coverage. Only the first operated eye from each eligible patient was included in the analysis.

Patients were excluded if they had prior diagnosis of uveitis or glaucoma, had lack of insurance coverage for the intracanalicular insert, or had insufficient data in the electronic medical record to determine inclusion/exclusion. Electronic medical records were reviewed to determine inclusion and exclusion eligibility for patients receiving the intracanalicular dexamethasone insert first, and after this, a similar number of patients receiving topical drops that met the same inclusion/exclusion criteria were consecutively screened. This ensured that the 2 consecutively screened series of charts included patient populations that were as similar as possible.

Dextenza (Ocular Therapeutix, Inc.) is a 0.4 mg dexamethasone ophthalmic insert designed to be placed into the canaliculus via the lower punctum. It is the first FDA-approved intracanalicular insert, and this route of administration allows for the tapered delivery of medication to the ocular surface without the need for eyedrops. Approved in November 2018 and June 2019, it is indicated for pain and ocular inflammation after ophthalmic surgery, respectively. It is activated with moisture and then releases dexamethasone to the ocular surface for up to 30 days. The carrier substrate is gradually resorbed over approximately 90 days, obviating the need for manual removal. In the patients who received topical eye drops for their postoperative inflammatory prophylaxis, a regimen of tapering prednisolone acetate 1% (1 mg/mL) 4 times a day, 3 times a day, 2 times a day, and 1 time a day for 1 week each was used. Patients with a diagnosis of diabetic retinopathy were also instructed to take ketorolac tromethamine 0.5% drops 4 times a day for 1 month after surgery, regardless of the treatment group. Patients were not prescribed topical nonsteroidal anti-inflammatory drops for any other indication. Patients in both groups received intracameral moxifloxacin intraoperatively.

After cataract surgery, patients were seen at 1 day and 1 week postoperatively. Patients were also scheduled for 1 month postoperative visits; however, some of these visits were rescheduled or delayed in the setting of the COVID-19 pandemic. For purposes of comparison between groups, eyes were not included for data analysis at the final postoperative visit if this visit was greater than 16 weeks after surgery. IOP was measured at each appointment using applanation tonometry (Tonopen, Reichert Technologies). Slit lamp examination was also performed at each visit. Pain scores (reported on a 1-to-10 scale) and conjunctival injection and anterior chamber reaction (as defined by the Standardization of Uveitis Nomenclature criteria) were abstracted during chart review of each case.^13^ For the purposes of this study, breakthrough inflammation was defined as trace or more anterior chamber reaction with subjective patient complaints such as pain and photosensitivity between 1 week and 16 weeks postoperatively. Clinically significant CME was defined as best-corrected Snellen visual acuity worse than 20/20, not explained by another ocular comorbidity, and confirmed with optical coherence tomography. Patients were also counseled on signs and symptoms of breakthrough inflammation and infection and provided with contact information to call if these signs and symptoms occurred. Any urgent messages during the follow-up period of up to 16 weeks after surgery, as well as any office visits resulting from these messages, were also reviewed. Primary end points were proportion of eyes with (1) breakthrough inflammation requiring escalation of anti-inflammatory therapy and (2) IOP increase ≥10 mm Hg at 4 to 16 weeks of follow-up. Secondary end points included incidence of intraoperative complications, CME, and infectious sequelae.

Before commencing the chart review, a sample size calculation was performed. Based on an assumed 3% incidence of breakthrough inflammation with topical drops and the desire to detect a difference greater than 10%, it was determined that 114 eyes would need to be included per group (alpha = 0.05, power = 80%).^14^ All variables were summarized prior to analysis. Data were reported as mean ± SD or as proportions of patients/eyes. Variables based on the patient such as demographic variables were compared between the 2 study groups using 2-sample *t* tests for means or chi-square tests for proportions. Statistical significance was assumed with *P* < .05, and all statistical analyses were performed with SAS software v. 9.4 (SAS Institute, Inc.).

## RESULTS

**Figure 1.**
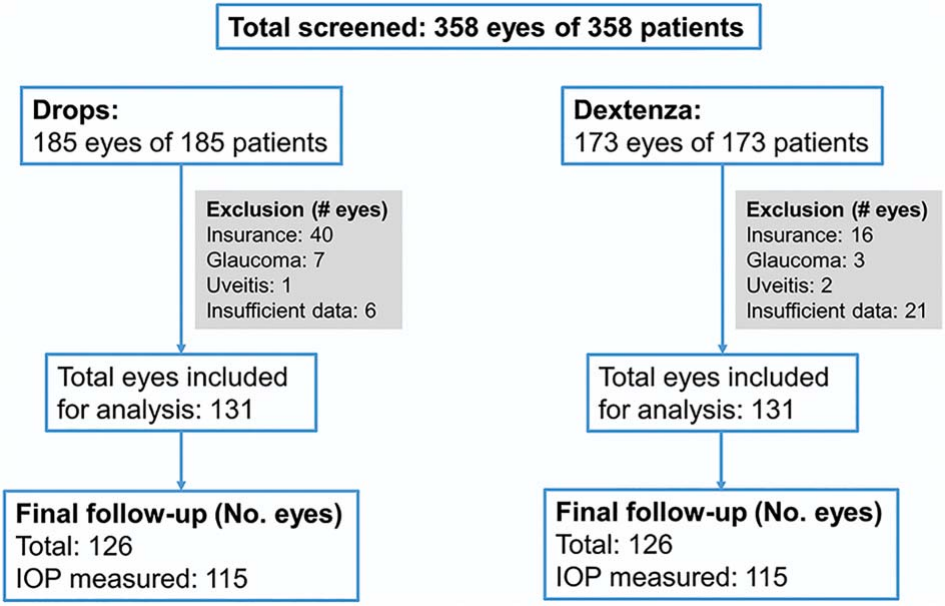
Patients and eyes that met the study inclusion/exclusion criteria.

A total of 358 eyes of 358 patients who underwent planned phacoemulsification cataract surgery by a single surgeon at the Penn State Eye Center between January 2018 and March 2021 were screened for eligibility. After screening, 262 eyes of 262 patients met the inclusion/exclusion criteria, and their electronic medical records were reviewed for data collection and statistical analysis (Figure 1). One hundred thirty-one eyes received topical drops, and 131 eyes received the intracanalicular insert. The drops group (n = 50, 38.2%) had a significantly higher proportion of patients with type 2 diabetes mellitus compared with the intracanalicular insert group (n = 35, 26.7%; *P* = .05; Table 1). However, the number of eyes that had diabetic retinopathy and received concurrent ketorolac tromethamine postoperatively was extremely similar between groups (drops: n = 16, insert: n = 13; *P* = .56). The drops group (n = 83, 63.4%) had a significantly higher proportion of patients with hypertension compared with the insert group (n = 65, 49.6%; *P* = .03; Table 1). There were no other differences between the drops and insert groups with regard to patient demographics, medical comorbidities, or baseline ocular characteristics (Tables 1 and 2).

**Table 1.** Patient demographics

Characteristic	Topical drops	Intracanalicular insert	*P* value
No. of eyes/patients	131	131	
Age (y)			
Mean ± SD	68.2 ± 10.0	68.7 ± 10.5	.74
Median	69	70	
Race, n (%)			
White	121 (93.1)	120 (94.5)	.87
Black/African American	2 (1.5)	3 (2.4)	
Asian	3 (2.3)	2 (1.6)	
Other	4 (3.1)	2 (1.6)	
Ethnicity, n (%)			
Hispanic/Latino	5 (3.8)	1 (0.8)	.21
Not Hispanic/Latino	126 (96.2)	123 (99.2)	
Medical comorbidities, n (%)			
Diabetes type 1	2 (1.5)	5 (3.8)	.45
Diabetes type 2	50 (38.2)	35 (26.7)	.05
Hypertension	83 (63.4)	65 (49.6)	.03
Autoimmune	29 (22.1)	19 (14.5)	.11

Race and ethnicity were not available in the electronic medical record of 5 and 7 patients, respectively

**Table 2.** Ocular demographics

Characteristic	Topical drops	Intracanalicular insert	*P* value
No. of eyes/patients	131	131	
Visual acuity (logMAR)			
Mean ± SD	.50 ± .44	.52 ± .46	.86
Snellen equivalent	20/63	20/63	
Baseline IOP (mm Hg)			
Mean ± SD	15.8 ± 3.1	16.0 ± 3.2	.68
Cataract, n (%)			
NS	101 (77.1)	108 (82.4)	.28
CS	74 (56.5)	60 (45.8)	.08
PSC	33 (25.2)	22 (16.8)	.10
Grading, mean ± SD	2.3 ± 0.5	2.2 ± 0.6	.14
Other ocular diagnosis, n (%)			
Epiretinal membrane	14 (10.7)	10 (7.6)	.39
Diabetic retinopathy	16 (12.2)	13 (9.9)	.56
Microvascular occlusion	2 (1.5)	3 (2.3)	>.99
Glaucoma suspect	17 (13.0)	17 (13.0)	>.99
Other	74 (56.5)	73 (55.7)	.90

CS = cortical; NS = nuclear sclerosis; PSC = posterior subcapsular

One hundred sixteen eyes in the drops group and 104 eyes in the insert group presented for 1-week follow-up. Of these, 113 eyes in the drops group and 102 eyes in the insert group had documented IOP measurement at 1 week. The mean change in IOP in the drops group was −0.8 ± 4.1 mm Hg; the mean change in IOP in the insert group was −0.4 ± 3.5 mm Hg (*P* = .49). At the 1-week examination, a higher proportion of eyes in the intracanalicular insert group (n = 48, 46.2%) had trace or more anterior chamber cell compared with those in the drops group (n = 37, 31.9%; *P* = .03). There was no difference between any reported pain (drops: n = 4, 3.5%; insert: 3, 2.9%; *P* > .99) or incidence of conjunctival injection (drops: n = 7, 6.0%; insert: 9, 8.8%; *P* = .43) between groups. There was no statistically significant difference in mean change in IOP (*P* = .70), any reported pain (*P* > .99), or incidence of conjunctival injection (*P* = .44) when the subset of eyes that received topical ketorolac was excluded. In subgroup analysis excluding eyes with diabetic retinopathy that received topical ketorolac, the intracanalicular insert group still trended toward a higher proportion of eyes with trace of more anterior chamber cell compared with eyes in the drops group, but this was no longer statistically significant (*P* = .11).

One hundred twenty-six of 131 eyes in each group presented for final follow-up. There were 2 cases of IOP elevation greater than or equal to 10 mm Hg in the drops group and 1 in the insert group. There was no difference in the mean change in IOP compared with baseline between groups (Table 3). Nine eyes (6.9%) in the drops group and 12 eyes (9.2%) in the insert group experienced breakthrough inflammation (*P* = .50). Among patients who received drops and experienced breakthrough inflammation, 4 admitted to poor compliance. In 1 additional case, the patient expressed extreme anxiety about touching her eyes, and the physician questioned the efficacy of drop administration. This patient experienced increasing photosensitivity and inflammation in the first week after surgery and eventually received a tap and inject to empirically cover for endophthalmitis. The culture was positive for *Propionibacterium acnes*, and inflammation slowly improved over the ensuing month. Of the 12 eyes in the insert group that experienced breakthrough inflammation, the insert could not be visualized in 2 cases. Twelve eyes (9.2%) in the drops group and 8 eyes (6.1%) in the insert group developed clinically significant CME (*P* = .35). There were no significant differences in pain, conjunctival injection, or anterior chamber reaction between the groups. Table 3 shows more detailed analysis comparing groups at the final visit. There were no cases of other severe adverse events such as suprachoroidal hemorrhage or retinal detachment in either group. There was also no statistically significant difference in breakthrough inflammation (*P* = .38), mean change in IOP (*P* = .93), CME (*P* = .39), pain (*P* = .37), conjunctival injection (*P* > .99), or anterior chamber cell (*P* = .91) between groups after eyes with diabetic retinopathy were excluded in subgroup analysis.

**Table 3.** Final postoperative visit

Characteristic	Topical drops	Intracanalicular insert	*P* value
No. of eyes/patients (total)	126	126	
No. completed slitlamp examination	115	115	
No. completed IOP check	113	104	
Breakthrough inflammation, n (%)	9 (6.9)	12 (9.2)	.50
IOP (mm Hg), mean ± SD			
Final IOP	15.0 ± 4.1	15.2 ± 3.2	.74
Change from baseline	−0.70 ± 4.7	−0.81 ± 3.6	.85
CME, n (%)	12 (9.2)	8 (6.1)	.35
Endophthalmitis, n (%)	1 (0.8)	0 (0.0)	>.99
Pain reporting, n (%)	3 (2.4)	1 (0.8)	.62
Conjunctival injection, n (%)	2 (1.8)	2 (1.9)	>.99
AC reaction, n (%)	7 (6.2)	7 (6.7)	.87

AC = anterior chamber; CME = cystoid macular edema

Among all eyes included in the study, patient demographics, diagnosis of systemic medical conditions, including diabetes mellitus, hypertension, and autoimmune disease, and diagnosis of other ocular conditions were not significantly associated with breakthrough inflammation (Table 4). Change in IOP, anterior chamber cell, and presence of conjunctival injection and pain at week 1 were significantly associated with breakthrough inflammation. Analysis of any factors associated with IOP elevation was deferred because of the very low incidence of this event in both study groups.

**Table 4.** Factors associated with breakthrough inflammation

Characteristic	*P* value
Age	.18
Race	.20
Ethnicity	.41
Comorbidities	
Diabetes type 1	.10
Diabetes type 2	.69
Hypertension	.69
Autoimmune	.13
Other ocular diagnosis	
Epiretinal membrane	.23
Diabetic retinopathy	.49
Microvascular occlusion	.34
Glaucoma suspect	>.99
Other	.14
Preop	
Visual acuity	.09
IOP	.18
NS	.40
CS	.43
PSC	.40
Cataract grade	.76
1-wk visit	
IOP change	**.03**
Pain	**.02**
Conjunctival injection	**.01**
AC reaction	**<.001**

AC = anterior chamber; CS = cortical; NS = nuclear sclerosis; PSC = posterior subcapsular

Bold values indicate *P* < .03

## DISCUSSION

Dropless cataract surgery can take many forms and may vary by the agent used and route of delivery. Each agent/route combination has its own profile of advantages and disadvantages, thus warranting independent investigation. For example, subconjunctival and sub-Tenon injection of steroidal agents has resulted in similar degrees of postoperative inflammation and lower IOP compared with topical drops.^15,16^ On the other hand, the effect of intraoperative sub-Tenon triamcinolone injection on IOP depends on the location of drug administration, with more anterior delivery being associated with higher IOP postoperatively.^17,18^ Transzonular and pars plana injection of triamcinolone has also been deemed successful; however, infrequent cases of endophthalmitis remind us that any perceived benefit to this route may be outweighed by the associated risks.^14,19,20^ A dexamethasone suspension, Dexycu (Eyepoint Pharmaceuticals, Inc.), gained FDA approval for intracameral delivery in 2018. It resulted in a 10.3% incidence of breakthrough inflammation, which was similar to the topical steroid control group.^21^ There was also a trend toward higher IOP in the intracameral group.

The dexamethasone-eluting intracanalicular insert is a unique addition to the above options for postoperative inflammation prophylaxis. The present study shows that it has an incidence of breakthrough inflammation well within the range reported across the literature (0% and 10.1%).^14,22^ Although there was a trend toward higher breakthrough inflammation in the insert group (9.2%) compared with the topical drops group (6.9%), this finding was not statistically significant (*P* = .50). It does not appear to increase IOP, and the risk for severe adverse events like endophthalmitis appears remote given its route of administration. If the intracanalicular insert were to cause a problem, it can be flushed from the canaliculus with balanced salt saline through a cannula. A secondary gain may be derived from the incidental occlusion of the punctum by the carrier substrate, which might leave the ocular surface better lubricated in the immediate postoperative period.

Like all therapeutic interventions, proper counseling on what to expect is essential. In the case of the intracanalicular insert, patients should be advised that they may note an increase of epiphora in the short term while the insert is resorbing. Although not specifically analyzed in this study, investigators have noted anecdotally that some patients reported increased tearing in the postoperative period. Most importantly, they should be counseled up front on the signs and symptoms of breakthrough inflammation, which include increased conjunctival hyperemia and photosensitivity. We found that this conversation was best presented on the day after cataract surgery. If done in this way, patients are educated on what to look for and when to call, and instances of breakthrough inflammation are less likely to result in after-hours or urgent encounters. Another point of counseling for patients is the potential financial burden of the intracanalicular insert compared with topical drops. The cost of the intracanalicular insert is approximately $500 to 600 USD, whereas the cost of a bottle of 1% prednisolone acetate or 0.05% difluprednate is $20 to 60 and $200 to 250 USD, respectively (price estimates from GoodRx.com, retrieved April 15, 22). In the present study, only patients whose insurance covered the insert were included in either group to minimize the possibility that differences in patients' insurance status indirectly led to other differences between the cohorts. It was noted during the data collection process that Medicare and most commercial insurance carriers covered the cost of the insert, whereas Medicaid did not. It is important that both patients who prefer the convenience of dropless cataract surgery, as well as those who have medically indicated difficulties with application of topical drops, be made aware of their individual insurance plans' coverage and the potential financial burden in the case of noncoverage.

In a post hoc analysis, several additional factors were identified that trended toward being predictive of breakthrough inflammation (Table 4). Patients with type 1 diabetes mellitus and/or autoimmune disorders and eyes with poor vision at baseline may benefit from closer monitoring for breakthrough inflammation. Clinicians might consider more aggressive inflammatory prophylaxis such as a combination of the intracanalicular insert with either topical steroid drops, topical NSAID drops, both, or a longer duration of anti-inflammatory therapy. Furthermore, eyes with anterior chamber cell, conjunctival injection, or reported pain at 1 week may benefit from more aggressive inflammatory prophylaxis as well (all *P* < .03; Table 4). Despite the correlation of anterior chamber cell at 1 week and breakthrough inflammation, it is interesting that the insert group had greater incidence of anterior chamber cell at 1 week, but this did not correlate with a significantly higher proportion of eyes having breakthrough inflammation in the insert group. This may be due to the insufficient number of eyes to power this post hoc analysis of risk factors associated with breakthrough. However, it may also indicate that the insert is associated with a mild amount of anterior chamber inflammation at 1 week but not to the extent that it becomes clinically significant. Another possibility is that not enough drug is released in the first few days after surgery in some patients, and if anterior chamber reaction is identified on examination at 1 week, additional anti-inflammatory agents are indicated—which would warrant further study. These data demonstrate that personalized approaches to prevention of breakthrough inflammation after cataract surgery may lead to improved safety and efficacy outcomes.

One limitation of the present study is its retrospective study design; as such, the patients included in the analysis were not randomized, and the investigators were not blinded. On the other hand, the chart review was performed consecutively to minimize the risk for reporter bias. In addition, the study was insufficiently powered to reveal differences in the proportion of eyes experiencing rare events like endophthalmitis. Some patients were excluded because of prior diagnosis of glaucoma (Figure 1). These patients are at a higher risk for developing IOP elevation on topical drops both within the first 24 hours and within the first few weeks after cataract extraction.^23,24^ Future work that allows for the randomization of subjects to a treatment arm and larger cohorts might be sufficiently powered to analyze subgroups such as those with glaucoma. Another implication of our finding that the intracanalicular dexamethasone insert is able to achieve comparable safety and efficacy to those of topical steroid drops after cataract surgery is that this approach may be broadened to other types of ophthalmic surgeries that have traditionally used topical drops to prevent postoperative inflammation. These include corneal transplantation and vitreoretinal surgery and represent exciting other avenues of future study.

**WHAT WAS KNOWN**

- Topical corticosteroids remain a mainstay for the prevention of postoperative inflammation after cataract surgery.
- A corticosteroid impregnated intracanalicular insert is superior to placebo in the prevention of postoperative inflammation after cataract surgery.

**WHAT THIS PAPER ADDS**

- The corticosteroid impregnated intracanalicular insert is similar to topical drops in its prevention of symptomatic breakthrough inflammation after cataract surgery.
- The incidence of steroid-induced ocular hypertension after cataract surgery may be considered a rare event, and use of the intracanalicular insert does not appear to increase this risk over the use of a tapering topical drop regimen.
